# Selective Polymer Distributed Bragg Reflector Vapor Sensors

**DOI:** 10.3390/polym10101161

**Published:** 2018-10-17

**Authors:** Paola Lova

**Affiliations:** Dipartimento di Chimica e Chimica Industriale, Università degli Studi di Genova, Via Dodecaneso 31, 16146 Genova, Italy; paola.lova@edu.unige.it

**Keywords:** photonic crystals sensors, polymers, distributed Bragg reflectors, Flory–Huggins, nanoparticles

## Abstract

We report on Flory–Huggins photonic sensors for the selective detection of volatile organic compounds without the use of any chemical functionalization. For this purpose, we employed periodic multilayers made of inert cellulose acetate alternated to active polystyrene films whose free volume was modified with silanized ZnO nanoparticles. The simple UV-visible (UV-vis) dynamic optical response of such polymer distributed Bragg reflectors during exposure to vapors of benzene, toluene, o-dichlorobenzene, and carbon tetrachloride allows their detection and recognition based on different chemico–physical affinity with the active polymer medium.

## 1. Introduction

The increasing production and consumption of goods is releasing harmful chemicals into atmosphere, water sources, and soil. Among these pollutants, volatile organic compounds (VOCs) pose serious environmental harm and concern to the human body. Table 1 provides a classification of common VOCs based on their effect on the human body. The list shows that, among others, commonly used solvents such as acetone can cause neurological damages, and that tetrachloroethylene, frequently used for dry cleaning, is a suspected carcinogenic agent [1,2]. Among other effects, indoor VOC pollution causes sick building syndrome [3], leading to eye and pulmonary irritation, headaches, loss of coordination, and nausea [4]. In the long-term, this exposure can damage the liver, kidneys, and central nervous system. VOC poisoning is also linked to the development of cancer [5]. Moreover, these pollutants contribute to the formation of the tropospheric ozone, a greenhouse gas, by reaction with oxygen sources such as nitrogen oxides and carbon monoxide [6]. The tropospheric ozone is contributing to global warming [7] and is a powerful oxidizing agent that causes respiratory illness [8], and can react with other chemicals to form new toxic pollutants [9]. VOCs are released by many industrial processes such as oil refineries, power plants, and chemical manufactures, and in urban areas by automotive vehicles, painting works, dry cleaning, refrigerators, wood burning, and photocopy machines [3,4,10]. The high level of toxicity and the wide spreading of these compounds make their identification and extensive monitoring in living and working environments critical to preserve people’s health and to identify proper treatments in the case of poisoning. Quantitative monitoring is commonly performed by portable detectors such as colorimetric tubes, metal oxide, and infrared sensors, which are very sensitive but lack of selectivity [11,12,13]. Qualitative assessment requires instead time-consuming sampling, chromatography, and detection using two or more detectors combined together [14]. These characteristics make the label-free photonic sensors described here a promising alternative [15,16].

Recently, photonic sensors based on the Flory–Huggins interaction parameter between analytes and all-polymer distributed Bragg reflectors (DBRs) entered in the spotlight for the development of label-free colorimetric sensors capable of broad selectivity among a large amount of analytes and that do not require specific chemical functionalization [17,18,19,20]. In these systems, DBRs made of alternated layers of two polymers with different refractive index [21] play as dense membranes for the intercalation of the analytes interacting selectively with them, thus allowing detection of any kind of permeating chemical species through the simple estimation of the Flory–Huggins parameter (χ). Moreover, thanks to low fabrication costs, ease of integration in lab-on-a-chip devices and of large area production using established technologies used in the food packaging industries [22,23,24,25,26,27], these DBR sensors are very promising for the development of safety devices and quali-quantitative detection systems.

In detail, the interaction between light and DBRs provide a typical optical response recognizable as a strong peak in their reflectance spectrum, also known as photonic band-gap (PBG) [28,29,30,31]. Such a peak arises from the coherent diffraction of incident light beams occurring at all the interfaces within the multilayer. The PBG can then be compared to the X-ray diffraction peaks arising from crystalline materials. Indeed, as the variation of lattice parameters in a crystal affects its diffraction pattern, variation in periodicity and refractive index of a DBR affects its reflectance spectrum. Then, when an analyte intercalates within the multilayer, swelling or shrinking the polymer chains, the photonic lattice, and in turn its spectrum, are modified. In sensing, the different chemico–physical affinity between analytes and polymers induces different intercalation kinetics, followed by the dynamic evolution of the DBR optical response. Indeed, the intercalation kinetics are strictly related to the polymer–analytes solubility, and then to the Flory–Huggins parameter for the couples [17], which has successfully been used to describe selectivity in polymer DBRs made by the alternated layer of a block-copolymer and poly(vinyl alcohol) [20]. This allows recognition of the analyte and its concentration [15]. Such systems have been proven to be sensitive to a variety of analytes including toluene [15], water [32], alcohols, and even perfluorinated compounds [17,33].

In a previous work, we demonstrated that DBRs containing phase changing polymers, which form a semicrystalline clathrate with different optical properties through analyte intercalation, allow label free selectivity among carbon tetrachloride (CTC), benzene (BEN), toluene (TOL), and 1,2-dichlorobenzene (o-DCB) [16]. In such a case, the selectivity was achieved by exploiting the different kinetic of crystallization of the active polymer when exposed to the analytes, and the specific porosity imprinted by the desorbed analytes within the active sensing medium. In this work, we show that the dynamic optical response of the DBR also allows the use of amorphous commodity polymers to recognize these VOCs. To this end, we used cellulose acetate (CA, *n* = 1.46) as low index and inert medium and a polystyrene (PS) nanocomposite doped with ZnO nanoparticles (ZnONP@PS, *n* = 1.59 [15]) as high refractive index and active sensing medium to detect and recognize TOL, BEN, o-DCB, and CTC. Indeed, CA has large free volume and permeability to gas and vapors, and its Flory–Huggins parameter ranges from 1.2 to 2.1 for the four analytes (Table 2), making it a an appropriate inert medium [15]. Conversely, PS permeability is usually low, but its Flory–Huggins parameter is usually very small for the chemical species under consideration, making it well suitable as active medium (Table 2). To increase its permeability, we doped the dense matrix with silanized nanoparticles to introduce modification in the polymer configuration at the ZnO–polystyrene interface [15]. This approach grants an increase of the polymer free-volume and then of the vapor permeability, allowing sensitivity below the part per million (ppm) and lower detection limit of ~20 ppm to toluene vapors [15]. This also allows faster response than those reported for bare polymer DBRs [15,17,20], but while maintaining the high processability typical of amorphous polymers, which is a key factor for scaled-up fabrications. Indeed, DBRs made of amorphous commodity polymers are currently the only photonic structure availing of industrial fabrication techniques [34]. This aspect, together with their colorimetric optical response, makes these systems interesting disposable sensors for air pollutants in industrial areas, as well as integrable transductors for detection of degradation by-products in goods packaging.

## 2. Materials and Methods

Synthesis and functionalization of ZnO nanoparticles (NPs): ZnONPs synthesis and silanization was run accordingly to previous reports [15,35]. The particles were synthetized via a solvothermal route from zinc acetate dihydrate and potassium hydroxide: 0.07 mol of zinc acetate were dissolved in methanol and heated at 63 °C under sonication. Once this temperature was reached, 0.14 mol of KOH was dissolved in the same solvent and slowly added to acetate solution. After 3 h of reaction under sonication, the particles were purified by five cycles of decantation and washing with methanol, and finally dried [36]. To avoid aggregation, dimethyl-(methoxy) octadecylsilane (DMMOS) was grafted onto their surface: 10 g of NPs was dispersed methanol and sonicated. Then, 70 mL of dichloromethane containing 2.5 g of DMMOS was added to the NP. The new dispersion was sonicated until complete evaporation and dried in a vacuum for 2 h. The graft reaction was then run at 135 °C under nitrogen flux for 2 h. 

Active polymer and DBR preparation: after silanization, the ZnONPs were dispersed into a toluene-polystyrene (MW = 200,000) solution and stirred. The new dispersion was used to grow DBRs alternating it to cellulose acetate (MW = 61,000) dissolved in 4-hydroxy-4-methylpentan-2-one (diacetone alcohol, DA). The polymer concentrations were 3% and 4% (*w*/*v*) for PS and CA, respectively. The rotation speed was 160 rounds per second. 

For all the DBR samples, reflectance data were collected with home-made setups based on optical fiber using a Y-fiber probe and an Avantes (Apeldoorn, The Netherlands) AvaSpec-2048 spectrometer (200−1150 nm, resolution 1.4 nm). The light source was a combined deuterium–halogen Micropak DH2000BAL (Ocean Optics, Largo, FL, USA).

Sensing measurements were performed as previously described [15] at 20 °C and 1 atm with the optical set-up described above using a dip-probe Y-fiber (FDP-7UVIR200-2-yy, Avantes, Apeldoorn, The Netherlands) where the sample was placed. The probe was then inserted and sealed in a glass tube where 500 µL of liquid analyte were previously placed to saturate the air environment. The distance between the sample and the liquid surface was kept at about 3 cm. The partial pressure for the four vapors in the given condition was calculated as follows: [37] carbon tetrachloride 11.95 kPa, benzene 10.5 kPa, 1,2-dichlorobenzene 0.13 kPa, and toluene 2.8 kPa (Table 2). 

## 3. Results and Discussion

**Figure 1:**
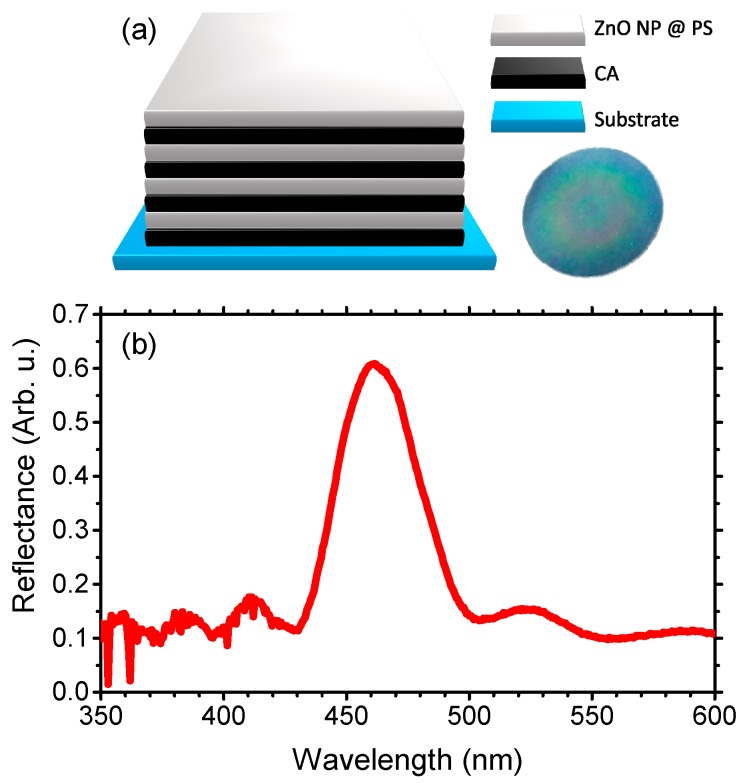
(**a**) Schematic and photograph of the ZnO nanoparticles (ZnONP)@PS/CA distributed Bragg reflector (DBR). (**b**) Reflectance spectrum of the sample.

The DBR sensors investigated in this work are made of 10 bilayers of CA and ZnONPs@PS nanocomposite supported on glass substrates. The samples appear blue, with the typical iridescence of photonic crystals (Figure 1a). In their reflectance spectrum, shown in Figure 1b, it is indeed possible to detect a maximum of intensity in the blue region of the visible spectrum at ~460 nm, which is assigned to the PBG, and provides the DBR with the blue color. In the spectrum background, an interference fringe pattern is observed, indicating the presence of well-defined external interfaces and an overall good optical quality [28,29,30]. 

**Figure 2:**
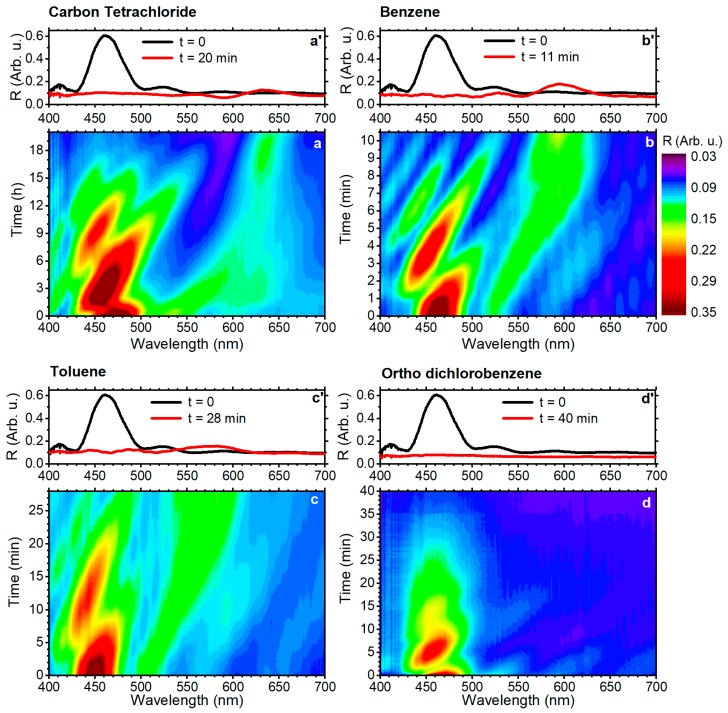
Optical response of the DBR sensors to vapors. The bottom panel reports the dynamic responses as contour plots (**a**–**d**). The top panels (**a’**–**d’**) show the spectra collected before and after the exposures for carbon tetrachloride (CTC) (**a**,**a’**), benzene (BEN) (**b**,**b’**), toluene (TOL) (**c**,**c’**), and 1,2-dichlorobenzene (o-DCB) (**d**,**d’**).

The DBR sample shown in Figure 1 was cut into different portions, and each of them was exposed to saturated vapor of BEN, TOL, o-DCB, and CTC in a close environment. Figure 2 shows the dynamic optical response of the sensors measured during the exposure to the four vapor analytes. The top panels (a’–d’) of the figure report the spectra collected before the exposure (black line) and after the equilibrium saturation is reached (red line). The bottom panels show instead the dynamic response over the entire exposure time as a contour plot (a–d). Here, the x-scale represents the wavelengths, while the y-axes report the exposure time. The spectra intensity is instead shown as color code from blue for lower reflectance, to red for higher values, as shown in the sidebar. 

Figure 2a,a’ displays the data collected during the exposure to CTC vapors. In this case, the PBG, positioned at 460 nm, undergoes several intensity oscillations until its intensity fades at ~16 min of exposure. Contemporarily, a new peak appears at 535 nm. This feature undergoes a monotone red-shift until 640 nm in ~18 min, when the response reaches the steady state. We also notice the intensity of this feature slightly fading in the time interval between 5 min and 15 min. This behavior has been previously observed for polymer DBR sensors and is the result of the progressive swelling of the DBR layers from the top one in contact with the vapor rich environment [15]. Indeed, the intensity of the PBG at 460 nm fades as a result of the swelling of the overlaying polymer layers, which do not contribute to the formation of the PBG anymore. Such swelling affects the total thickness of the DBR, and in turn dynamically affects the interference pattern. The spectral interference between the Fabry–Perot pattern and the PBG generated the intensity and spectral oscillation detected in the PBG spectral region. The feature arising at 535 nm is instead assigned to the PBG of the swollen layers. It is indeed located at longer wavelength (larger layer thicknesses) than the original PBG and undergoes further red-shift while the layers keep swelling. 

When exposed to BEN and TOL, samples show a similar behavior, but in the case of benzene, the PBG of the swollen structure is detectable after 11 min at ~600 nm (Figure 2b,b’), while for toluene, the kinetic is slower, and the PBG reaches 550 nm in the same amount of time, while it requires double the time of that required by BEN to reach the steady state (Figure 2c,c’). o-DCB provides a further different response (Figure 2d,d’). In this response, the PBG observed at 460 nm shows the intensity oscillation previously observed, and completely fades within 35 min of vapor exposure. On the other hand, the spectral feature assigned to the swollen DBR sensor cannot be detected clearly. This particular effect has been previously assigned to the large steric hindrance of this molecule, which induces severe swelling as well strain and disorder in the DBR, which break its periodicity and then hinder the formation of the PBG [16]. 

**Figure 3:**
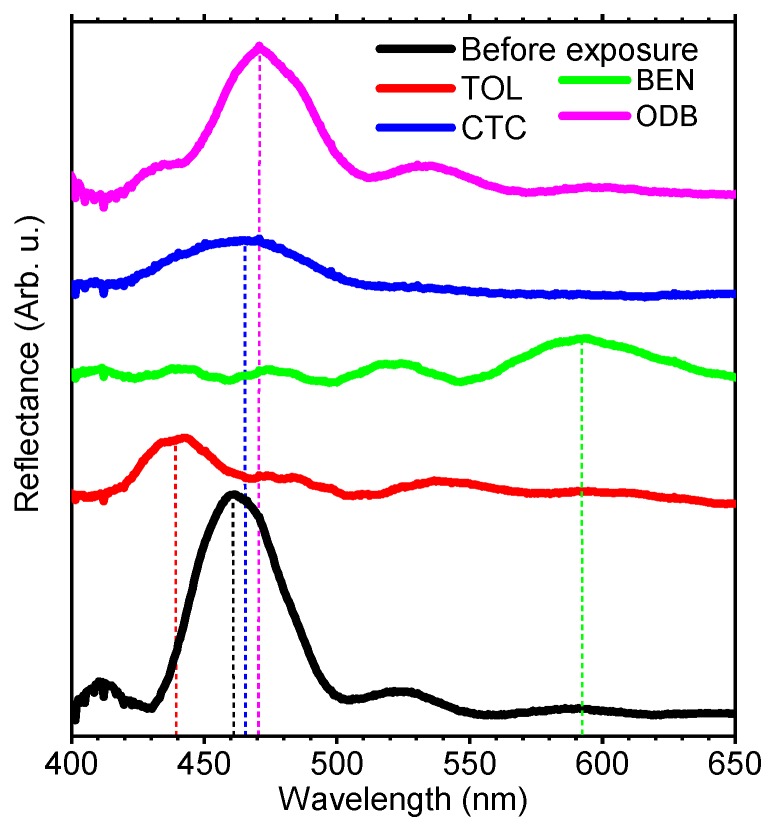
Reflectance spectra of ZnONP@PS @CA DBRs collected before (black line) and after 10 min of exposure to vapors of TOL (red), BEN (green), CTC (blue), o-DCB (magenta).

The very different optical response allows us to simply recognize the analytes. Indeed, in setting an arbitrary response time, it is possible to distinguish the analyte by the different spectral position of the PBG. To this end, Figure 3 compares the spectra of the sensors collected after 10 min of exposure to the vapors. Here, we notice that while the fingerprint of the response for TOL and BEN is similar (compares Figure 2b,c), their very different dynamic allows their recognitions. Indeed, after 10 min, toluene only induces a decrease in intensity of the PBG, which appears slightly blue-shifted with respect to its initial position (compare red and black lines in Figure 3). Conversely, the same exposure time allows the formation of a PBG assigned to the swollen structure when benzene is used. In this case, the PBG appears indeed at ~590 nm. CTC and o-DCB instead induce shifts of ~20 nm and ~30 nm respectively. These data demonstrate that the sensors response time can be as low as 10 min. Moreover, the literature shows that engineering the DBR layer thickness and the nanoparticle volume fraction, it is also possible to achieve faster responses [15,17,20]. Concerning the reversibility of the sensors, a previous report on a similar system [15] demonstrated that the sensor shows full spectral reversibility upon several cycles of analyte exposure and desorption, but the response velocity increases linearly with the number of exposures. Despite the lack of full reversibility, as mentioned above, these systems are interesting disposable devices thanks to available industrial fabrications at the square-meter scale [25].

Table 2 compares the optical shift of the PBG and the time required to reach saturation retrieved for the four VOCs with their van der Waals volumes, and Flory–Huggins interaction parameters. We notice that for the benzene derivates (BEN, TOL, and o-DCB), the values of Δλ_eq_ and t_eq_ increase with the analyte volumes and with **χ_PS_**, while no correlation appears with the interaction parameter calculated for CA, confirming that the PS matrix acts as active sensing medium. No correlation between the analyte concentration and the time or spectral shift can be evinced, suggesting that the selective response is independent from these parameters. Concerning CTC, both the PBG spectral shift at saturation and saturation time do not follow the same trend, and the response time is up to 100 times slower than for benzene derivates, while the spectral shift is lower than the one induced by these molecules, which have larger van der Waals volumes. Such behavior has already been observed in the literature and can be ascribed to different intermolecular forces instauration with the two polymers with respect to the benzene derivates [16]. 

## 4. Conclusions

This work demonstrates DBR sensors made of commodity polymers with high optical responsivity and selectivity to benzene, toluene, 1,2-dichlorobenzene, and carbon tetrachloride vapor exposure. The sensitivity to vapor was achieved by increasing the overall DBR permeability using a polystyrene–ZnO nanocomposite, while selectivity is achieved by analyzing the dynamic optical response of the sensors, which is affected by the different chemico–physical affinity between the active sensing polymer in the DBRs and the four VOCs. Together with the possibility to fabricate polymer DBR sensors on the square-meters area, these results pave the path to new low-cost disposable devices for safety purposes to be employed in indoor and outdoor industrial environments. 

## Figures and Tables

**Table 1:** List of common volatile organic compounds (VOCs) based on their effect on the human body.

Effects	Compounds
Toxic to organ systems [1]	Toluene, xylenes, chlorobenzene, dichlorobenzenes, styrene, carbon disulfide, acrolein, chloroform, bromoform, 2-butanone, 1,3-butadiene, tetrachloroethane, dichloroethene, chloroethane, dichloropropenes, bromomethan, hydrazines.
Mutagen and developmental [1]	Xylenes, n-hexane, ethylene glycol, vinyl chloride, acrylonitrile, acrylamide, ethylbenzene, chloroform, chloroethane, dichlorobenzenes, phalates, ethylene oxide.
Neurological [1]	Fuels and mineral oils, toluene, acetone, n-hexane, benzene, xylenes, acrylonitrile, pyridine, trichloroethane, carbon tetrachloride, chloroform, 1,3-butadiene, ethylbenzene, mercaptanes, naphtalenes.
Potential carcinogen [2]	Polycyclic aromatic hydrocarbons, acrylonitrile, acrylamide, nitrobenzene, styrene, hydrazines, naphtalenes, halogenated hydrocarbons (e.g., chloroform, carbon tetrachloride, dichlorobenzenes, trichloroethylene, hexachloroethane polyhalogenated biphenyles)
Carcinogen [2]	Benzene, formaldehyde, vinyl chloride, ethylene oxide, benzidine, 1,3-butadiene, bis(chloromethyl) ether.

**Table 2:** Analytes van der Waals Volumes (V); Flory–Huggins parameter for PS (χ_PS_), CA (χ_CA_), and for the entire DBR (χ_eff_); photonic band-gap (PBG) spectral shift at the equilibrium (Δλ_eq_); time required to reach saturation (t_eq_); and analyte vapor pressure.

Analyte	V (Å2) [16]	χ_PS_ [38]	χ_CA_ [38]	χ_eff_ [38]	Δλ_eq_ (nm)	t_eq_ (min)	Vapor Pressure (kPa)
benzene	89.4	0.001	1.635	1.183	130	11	10.5
toluene	106.8	0.006	2.090	1.512	120	28	2.8
o-dichlorobenzene	112.8	0.088	1.223	0.887	--	40	0.13
carbon tetrachloride	97.1	0.008	1.942	1.406	175	>1200	11.95
